# In Situ Analysis of Binder Degradation during Catalyst-Accelerated Stress Test of Polymer Electrolyte Membrane Fuel Cells

**DOI:** 10.3390/ma17174425

**Published:** 2024-09-09

**Authors:** Donggeun Yoo, Sujung Park, Sohyeong Oh, Minsoo P. Kim, Kwonpil Park

**Affiliations:** Department of Chemical Engineering, Sunchon National University, Suncheon 57922, Republic of Korea; ehdrms6832@gmail.com (D.Y.); psj7425420@naver.com (S.P.); ohso45@naver.com (S.O.)

**Keywords:** PEMFC, binder, AST, degradation, DRT

## Abstract

High-oxygen-permeability ionomers (HOPIs) are being actively developed to enhance the performance and durability of high-power polymer electrolyte membrane fuel cells (PEMFCs). While methods for evaluating binder performance are well-established, techniques for assessing binder durability and measuring its degradation in situ during the AST process remain limited. This study examines the distribution of relaxation times (DRT) and Warburg-like response (WLR) methods as in situ analysis techniques during the catalyst-accelerated stress test (AST) process. We conducted catalyst-ASTs (0.6–0.95 V cycling) for 20,000 cycles, monitoring changes using cyclic voltammetry (CV), electrochemical impedance spectroscopy (EIS), and linear sweep voltammetry (LSV). Contrary to expectations, during the catalyst-AST, the ion transport resistance of the binder decreased, indicating no binder degradation. Scanning electron microscopy/energy dispersive spectrometer (SEM/EDS) analysis revealed that the degradation rate of the catalyst and the support was relatively higher than that of the binder, leading to a reduction in catalyst layer thickness and improved binder network formation. By applying the DRT method during the catalyst-AST process, we were able to measure the increase in oxygen reduction reaction (ORR) resistance and the decrease in proton transport resistance in situ. This allowed for the real-time detection of the reduction in catalyst layer thickness and improvements in ionomer networks due to catalyst and support degradation. These findings provide new insights into the complex interplay between catalyst degradation and binder performance, contributing to the development of more durable PEMFC components.

## 1. Introduction

Polymer electrolyte membrane fuel cells (PEMFCs) have been recognized as the most suitable energy conversion device for next-generation zero-emission Electric Vehicles (ZEVs). However, cost reduction and lifespan extension are essential for expanding the commercialization of PEMFCs [1,2]. To reduce stack cost, Pt alloys with high activity levels have been developed to lower cathode Pt loading to less than 0.1 mg/cm^2^ [3,4,5]. However, many studies have reported that low-Pt-loading catalysts show an increase in local oxygen resistance at high current densities [6,7,8,9,10,11]. Therefore, to improve the performance and durability at high current densities, high-oxygen-permeation ionomers (HOPIs) have been researched and developed [12,13,14,15,16,17,18]. The required lifespan for light-duty vehicles (LDV) and light truck ZEVs is 5000 to 8000 h [19], whereas high-duty vehicles (HDV) require a lifespan of approximately 30,000 h. Recently, the performance and durability of PEMFCs at high power have been emphasized more, as the demand for improved durability has increased with the focus on large truck ZEVs. Consequently, the performance and durability of binders have become increasingly important.

Many studies on the degradation and durability of catalysts and supports in electrode layers have been conducted, but research on the degradation and durability of binder ionomers is lacking [15,20,21]. The role of ionomers in catalyst layers is as follows: (1) the attachment of Pt to a membrane as a binder; (2) the transportation of hydrogen ions in catalyst layers. In addition, the binder ionomer should have oxygen permeability to facilitate oxygen transfer to the catalyst surface. The binder ionomer can decay due to chemical degradation and mechanical degradation, but the proton transfer resistance and oxygen transfer resistance of the binder ionomer can vary depending on the distribution of the ionomer in the catalyst layer. The distribution of the ionomer in the catalyst layer is affected by the amount and distribution of Pt and carbon. Namely, the performance of binder ionomers is influenced by electrode degradation, including the catalyst degradation and carbon support degradation.

Binder ionomers play a crucial role in the electrode catalyst layer of PEMFCs, binding catalyst particles, providing proton conductivity, and regulating reactant gas permeation. Recent research has focused on developing high-oxygen-permeability ionomers (HOPI) to enhance PEMFC performance [17,22,23,24,25]. However, while methods for evaluating binder performance are well-established, techniques for assessing binder durability and quantifying its degradation in situ during the AST process remain limited.

Ionomer degradation has been studied extensively, mainly in membranes. The methods of durability evaluation for polymer membranes include the chemical durability evaluation method (OCV holding), the Mechanical Durability evaluation method (Wet/Dry test), and the combined accelerated stress test (AST) method, which combines these two approaches [26]. Even though it is the same ionomer, these AST methods cannot be uniformly applied to evaluate the binder’s durability. In the case of polymer membranes, the durability targets according to DOE include OCV reduction, hydrogen crossover current density (HCCD), and short resistance [26]. These targets are values that can be electrochemically measured in situ. However, the durability of binders cannot be evaluated using these targets, even if the same ionomer is used. Therefore, binder durability has sometimes been assessed by changes in performance through I–V curves [26,27], but this method does not accurately evaluate binder durability, since it is influenced by other factors. Binder durability is also evaluated using the catalyst-AST [15,28], but this cannot be considered an accurate evaluation method for binder durability due to the degradation of catalysts and supports. During the catalyst-AST process, the catalyst Pt degrades, leading to a decrease in the electrochemical surface area (ECSA), and it is impossible to exclude the influence of the binder in contact with the catalyst. In other words, since the catalyst layer consists of the catalyst, the support, and a small amount of the binder in close proximity, it is difficult to isolate the binder and analyze its degradation and durability separately.

To assess binder degradation, analyses such as NMR [21,29], X-ray diffraction (XRD) [30], X-ray photoelectron spectroscopy (XPS) [31,32], and atomic force microscopy (AFM) [22,33,34] have been used. However, these methods are ex situ, meaning they cannot monitor the degree of degradation during the evaluation process. Morawietz et al. [34] reported analyzing the ionomer structure in the catalyst layer in situ, but this actually refers to an analysis performed under conditions with RH and temperatures similar to those of a cell, not in an actual operating cell. There is an impedance analysis method (EIS) that measures ion transport resistance in binders without disassembling the cell during operation, known as the Warburg-like response (WLR) [27,35]. However, after degradation, the binder’s resistance decreases, raising questions about whether this method accurately evaluates binder durability [27]. 

The objective of this study is to develop and validate methods for the real-time analysis of binder degradation during the catalyst-AST process. We apply the distribution of relaxation times (DRT) method and the Warburg-like response (WLR) method to measure changes in the binder’s ionic conductivity in real-time. Additionally, we compare these electrochemical analysis results with ex–situ analysis using SEM–EDS to verify their reliability. Through this approach, we aim to accurately assess changes in binder performance during the catalyst-AST process and understand how alterations in catalyst layer structure affect binder performance. This information will be crucial for the design and optimization of binders to improve PEMFC performance and durability.

## 2. Materials and Methods

### 2.1. Cell Clamping and Operation Condition

A membrane electrode assembly (MEA) was fabricated by hot pressing a Pt/C catalyst at a loading of 0.4 mg/cm^2^ onto both the anode and cathode of a Nafion 211 (dupont, Wilmingon, NC, USA) membrane. The MEA was clamped to an active area of 25 cm^2^, using a gas diffusion layer (sigracet 39BB, SGL carbon, Wiesbaden, Germany) and a carbon flow field (five-channel, serpentine).

### 2.2. Catalyst-AST Protocol

Catalyst durability evaluation was performed based on the DOE catalyst-accelerated degradation protocol [26]. The cell was subjected to a square wave potential change of 0.65–0.9 V for 3 s using a potentiostat (HCP 805, biologic, Claix, France). During the durability evaluation, 200 mL/min of hydrogen was supplied to the anode of the cell, and 80 mL/min of nitrogen was supplied to the cathode, and the cell temperature and humidity were maintained at 80 °C and RH 100%.

### 2.3. Electrochemical Characterization

The I–V polarization curve was conducted at atmospheric pressure, supplying hydrogen at 500 mL/min to the anode and air at 2200 mL/min to cathode. The open circuit voltage (OCV) was measured by supplying hydrogen at 93 mL/min to the anode and air at 296 mL/min to cathode.

CV measurements were conducted for 15 cycles at a scan rate of 30 mV/s in the potential range of 0.05–1.2 V. The electrochemical surface area (ECSA) is calculated by using the formula of $ECSA=Q_{H}/\left(L\times q_{H}\right)$, where $Q_{H}$ is the hydrogen adsorption charge, L is the mass of platinum loaded on the electrode (mg/cm^2^), and $q_{H}$ is the charge required to oxidize a monolayer of hydrogen on platinum (0.21 mC/cm^2^). Specifically, the ECSA was determined by integrating the current density from the 0–0.4 V hydrogen desorption region, using the area above the trend line in the 0.4 V electric double-layer region.

LSV measurements were performed in the potential range of 0–0.5 V at a scan rate of 1 mV/s, with a potential range of 0–0.5 V. The hydrogen crossover current density (HCCD) is an oxidation current of hydrogen that has passed through the electrolyte membrane from the anode to the cathode. The current density value at 0.3 V was considered the HCCD. The short resistance (SR) was calculated to range from 0.4 to 0.5 V using Ohm’s law.

EIS was measured using an impedance analyzer (SI 1260, AMETEC Inc., Chester, PA, USA). EIS was carried out at 40 mA/cm^2^. The frequency of the sinusoidal current perturbation signal varied from 0.01 Hz to 100 kHz, using an amplitude of 10% of the current set point. 

The WLR impedance was measured by supplying 200 mL/min of hydrogen to the anode and 200 mL/min of nitrogen to the cathode, while applying a 5 mV perturbation at OCV over a frequency range of 0.01 Hz to 100 kHz.

### 2.4. Distribution of Relaxation Times 

The mathematical relation between the complex impedance *Z*(*ω*) and the distribution function of relaxation times *g*(*τ*) is shown in (1) [36,37]:

(1) $$Z\left(\omega \right)=R_{0}+R_{pol}\int_{0}^{\infty }\frac{g(\tau )}{1+i\omega \tau }d\tau$$

$R_{0}$, $R_{pol}$, and *ω* represent the ohmic resistance, the polarization resistance, and the angular velocity (where ω=2πf, with f being the frequency), respectively. The time constant *τ* = RC. The measured EIS data are turned into distributions of the time constants using DRT tools [38]. To make the distribution function meaningful, the regularization parameter λ was set to 10^–5^ [39].

### 2.5. SEM–EDS

The cross-sections of the MEA and the element inside the membrane were analyzed using SEM–EDS (JSM–7100F, JEOL, Tokyo, Japan). To prepare a cross-sectional sample of the MEA, the MEA was immersed in liquid nitrogen for 10 min and then bent to break it. The MEAs were coated with Au and observed under a beam potential range of 5–15 kV. SEM images were obtained at 4000× magnification.

## 3. Results and Discussion

### 3.1. Electrochemical Characterization during Catalyst-AST

#### 3.1.1. Cyclic Voltammetry

**Figure 1:**
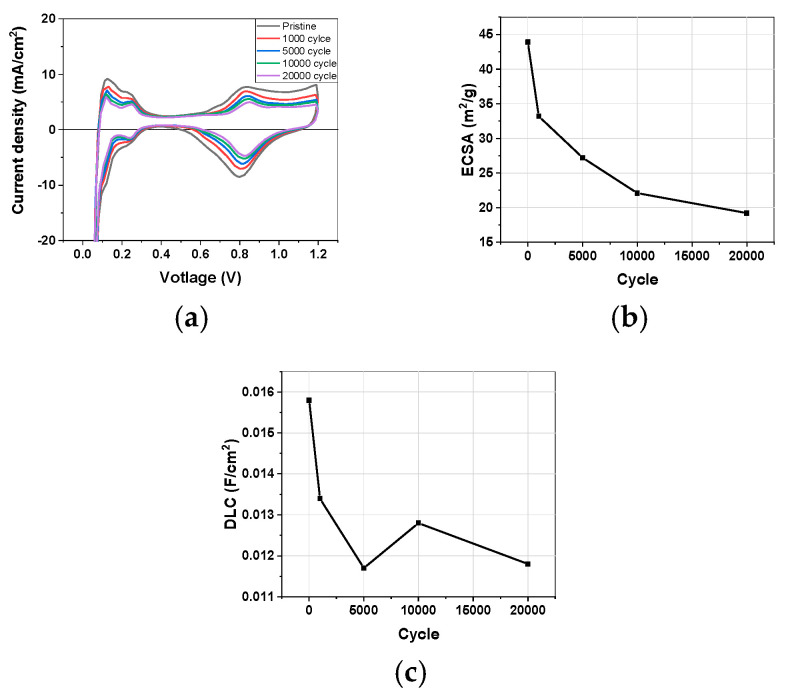
(**a**) CV changes during AST. (**b**) ECSA and (**c**) DLC changes for AST cycles.

Figure 1 shows the changes in the ECSA and DLC calculated from CV measurements during the Pt catalyst-AST process. Overall, a significant degradation of the cathode occurred during the 1000 cycles. The ECSA decreased by approximately 40% after 5000 cycles, reaching the DOE target (Figure 1b). Although the DOE AST is typically conducted up to 30,000 cycles, the experiment was terminated after 20,000 cycles as the ECSA had decreased to 56%. The ECSA mainly represents the catalyst’s active surface area, while the degradation of the carbon support can be identified through changes in the double-layer capacitance (DLC) (Figure 1c). The DLC, calculated from the central area of the CV curve, decreased by about 15% after 1000 cycles, indicating that the carbon support was degrading even at low voltages below 1.0 V [40]. After 20,000 cycles, the DLC had decreased by 25%, showing that, although not as much as that of the Pt, the degradation of the carbon support also significantly contributed to the overall degradation of the cathode.

#### 3.1.2. Impedance

**Figure 2:**
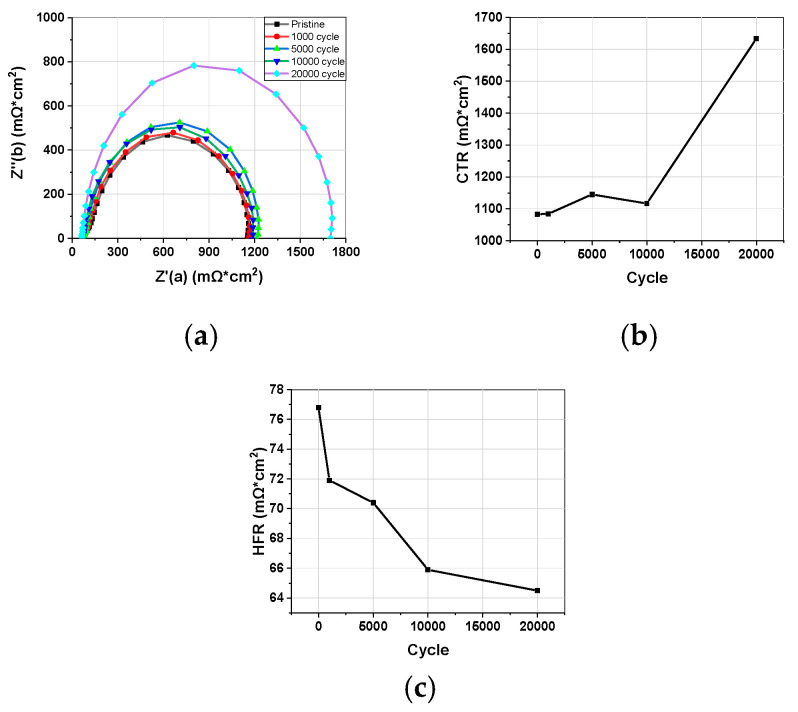
(**a**) Impedance changes during AST. (**b**) CTR and (**c**) HFR changes for AST cycles.

**Figure 3:**
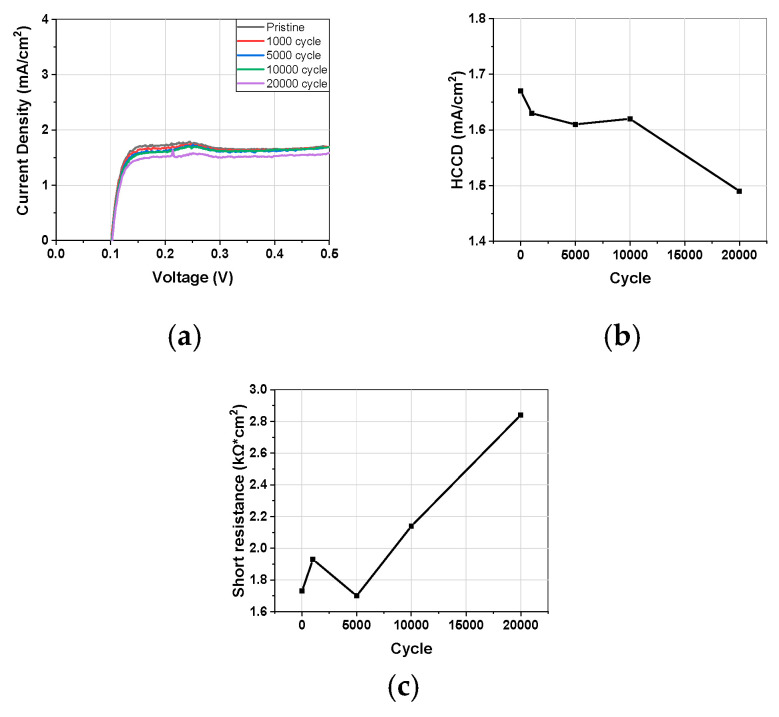
(**a**) LSV changes during AST. (**b**) HCCD and (**c**) short resistance changes for AST cycles.

**Figure 4:**
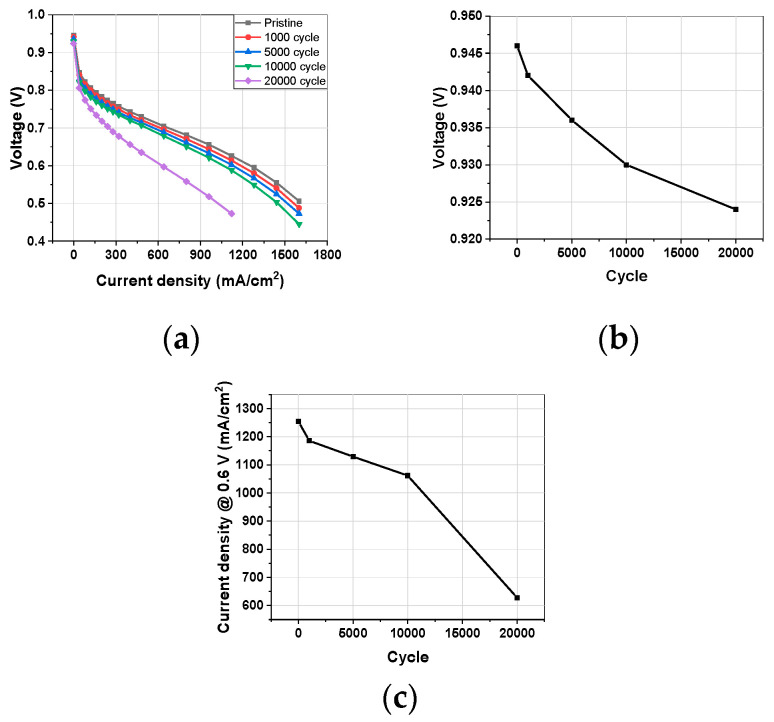
(**a**) I–V curve changes during AST. (**b**) OCV and (**c**) current density @ 0.6 V changes for AST cycles.

Impedance was measured during the AST, and the HFR and CTR changes are shown in Figure 2. As AST progressed, the overall impedance semicircle tended to increase. The HFR, which represents the membrane and membrane/electrode interface resistance, actually decreased, confirming that there was no deterioration of the polymer membrane (Figure 3c). The CTR, which represents the reaction resistance of the cathode, gradually increased up to 10,000 cycles, and then rapidly increased to 20,000 cycles (Figure 3b). Compared to the rapid decrease in ECSA and DLC at the beginning, there is a difference in that the CTR rapidly increased by 50.9% between 10,000 and 20,000 cycles. When comparing the CV results and the impedance results, the decrease in I–V performance and the increase in the CTR of the impedance are more consistent (Figure 4).

#### 3.1.3. Linear Sweep Voltammetry

To assess membrane degradation, LSV measurements were taken to calculate HCCD and short resistance, as shown in Figure 3. The HCCD gradually decreased during the AST process, showing about a 10% reduction after 20,000 cycles. This decrease in HCCD is attributed to catalyst degradation, specifically the decrease in hydrogen oxidation current density at the cathode due to the decrease in the Pt active surface area [41]. Normally, when the membrane degrades and its thickness decreases, the HCCD would increase, but in this case, since there was no reduction in membrane thickness, the HCCD actually decreased (Figure 2b). Catalyst degradation can lead to Pt dissolution and precipitation within the membrane, which might reduce short resistance. However, short resistance actually increased, indicating that the overall MEA performance reduction due to changes in short resistance is minimal (Figure 3c).

#### 3.1.4. Polarization Curve

Figure 4 shows the changes in I–V performance, OCV, and current density at 0.6 V during the AST. Overall, the I–V performance gradually decreased, with a sharp decline occurring between 10,000 and 20,000 cycles. This trend is consistent with the CTR, indicating that the reduction in I–V performance is primarily due to catalyst degradation. The OCV also gradually decreased, which suggests that the decline is not due to the membrane, since HCCD decreased and short resistance increased. Instead, it is attributed to the degradation of the electrode catalyst (Figure 4b).

### 3.2. Change in Binder Resistance 

#### 3.2.1. Distribution Relaxation Times

**Figure 5:**
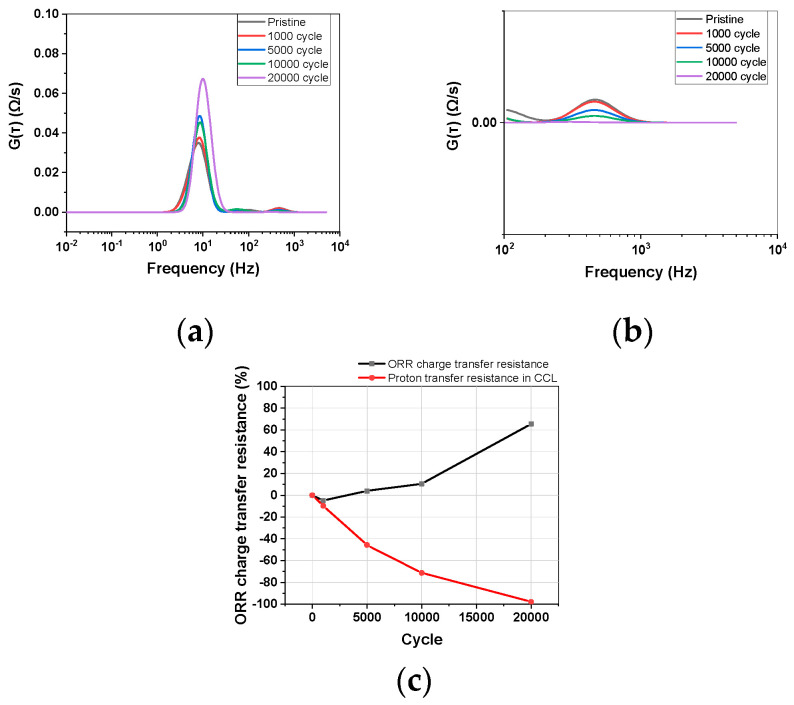
(**a**) DRT changes during AST. (**b**) Magnification of proton transfer resistance peak. (**c**) ORR charge transfer resistance and proton transfer resistance in CCL changes for AST cycles.

The results of impedance measurements during the catalyst-AST were analyzed using the DRT method and are presented in Figure 5. In Equation (1), g(τ) is dimensionless, but G(τ) is the product of R_pol_ and g(τ), with units of Ω/s. The Kramers–Kronig validation of each impedance data set showed that the residual was within 1% (Figure S1). As the catalyst-AST progressed, the catalyst degraded, leading to a decrease in ECSA and an increase in CTR, which was reflected in the increase in ORR resistance. The DRT method aligned well with these analysis results. However, the proton transport resistance of the binder ionomer decreased, suggesting that there was no degradation of the binder (Figure 5c). Although the catalyst-AST was conducted, it was expected that ionomer degradation would occur due to the high voltage and temperature, but the results showed the opposite. Other researchers have also evaluated binder durability using catalyst-AST (or load cycling, square wave cycling) and compared the durability with that of standard binders [15,17,27]. Emmauel et al. [20] compared binder durability based on CTR changes using EIS Nyquist plots, but since CTR is significantly influenced by the catalyst and catalyst support, it cannot be considered an accurate evaluation method. The idea behind using the catalyst-AST as a binder AST was based on the assumption that since the catalyst layer contains the binder ionomer, degrading the catalyst would also degrade the binder ionomer. However, the DRT results showed that the binder did not degrade, and, in fact, its performance (ionic conductivity) improved. Further analysis using other methods was conducted to investigate the reasons behind these results (Section 3.3.1).

#### 3.2.2. Warburg Like Resistance

**Figure 6:**
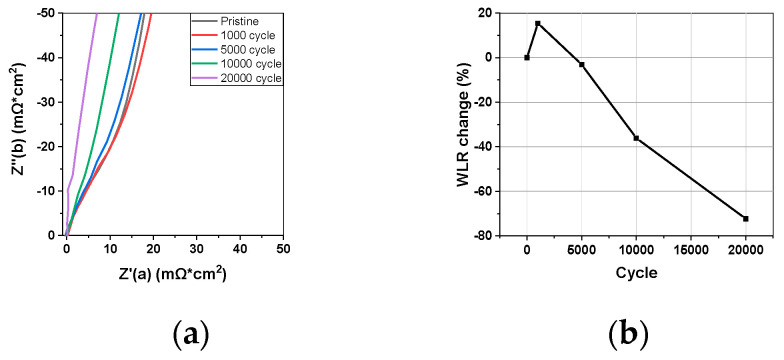
(**a**) WLR impedance changes during AST. (**b**) WLR changes for AST cycles.

The results of H_2_/N_2_ impedance measurements during the Pt catalyst-AST process were analyzed using the WLR method and are presented in Figure 6. Since no oxygen was supplied to the cathode, there was no ORR, and the resistance to proton transport in the binder was measured as the hydrogen supplied to the anode crossed over and was oxidized at the cathode. As the AST progressed, the WLR decreased, which was consistent with the DRT analysis results (Figure 6b). In other words, the proton conductivity of the binder increased. The same result was obtained from both impedance methods. Litster et al. [27] also reported a similar finding, with the WLR decreasing after the catalyst-AST.

### 3.3. Analysis of Catalyst Layer Degradation 

#### 3.3.1. Cross-Sectional SEM of MEA

**Figure 7:**
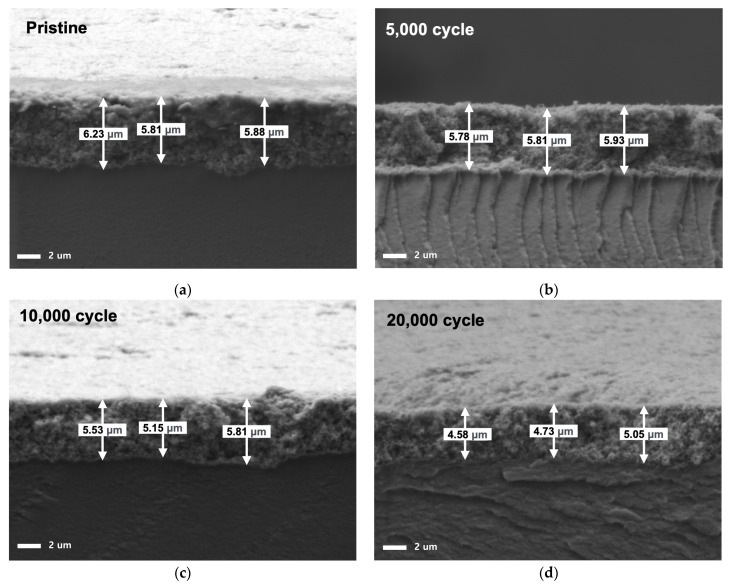
Cross-sectional SEM images of MEA after different AST cycles: (**a**) pristine, (**b**) after 5000 AST cycles, (**c**) after 10,000 AST cycles, and (**d**) after 20,000 AST cycles.

The degradation of the catalyst layer led to a reduction in cathode thickness, with the thickness decreasing by 19.1% after 10,000 cycles and 22.4% after 20,000 cycles (Figure 7). The Pt catalyst dissolved, causing Pt ions to be expelled into the membrane and GDL, reducing the amount of Pt, while the carbon support reacted with H_2_O, forming CO_2_, which was expelled along with nitrogen, leading to a decrease in thickness [40,41]. The binder ionomer also likely contributed to the reduction in cathode thickness due to degradation from radical attacks, similar to what occurs in the polymer membrane. However, ionomer degradation in polymer membranes is typically caused by HO·radicals, which require the presence of oxygen to form. Since the conditions involve nitrogen inflow, oxygen generation would be possible only through a water electrolysis reaction, but the voltage is too low for this reaction to occur. It has been suggested that the ionomer in both the membrane and binder could be degraded by H·radicals [21,42] and it is believed that hydrogen crossing over through the membrane formed radicals on the cathode Pt, leading to binder degradation.

#### 3.3.2. EDS of Catalyst Layer

**Figure 8:**
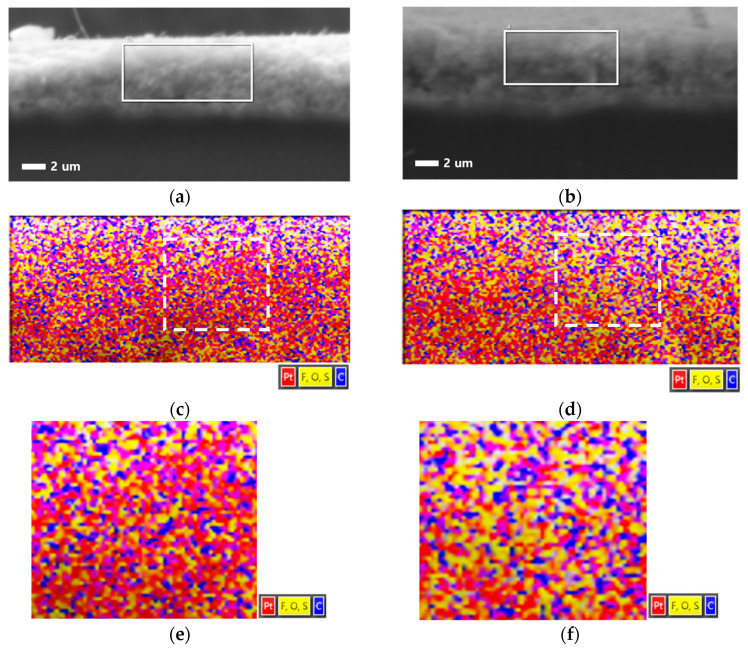
Cross-sectional SEM images of cathode catalyst layers: (**a**) pristine and (**b**) after 20,000 AST cycles. EDS mapping of the rectangular area within the cathode catalyst layer in the SEM image: (**c**) pristine and (**c**) after 20,000 AST cycles. Magnified images of the rectangular areas in (**c**) and (**d**) are shown in (**e**) and (**f**), respectively.

The cathode cross-section before and after the catalyst-AST was analyzed using SEM–EDS, and the results are compared in Figure 8. The elements of the binder ionomer, F, O, and S are shown in yellow, while the catalyst Pt is shown in red. Carbon, which is a component of both the carbon support and the binder, is shown in blue, although it is unclear which specific part it belongs to. Blue areas near yellow likely represent binder carbon, while blue areas near red are likely support carbon. Similar to how binder distribution was confirmed by measuring adhesion with AFM [22,33,34], SEM–EDS mapping can also show the distribution of the binder. Overall, after the AST, the amount of Pt decreased and the relative amount of binder increased. There is also an increase in blue near yellow, suggesting more organic carbon from the binder. The yellow (binder) network appears to be better connected after the AST compared to before. Although there was likely some binder degradation during the AST, the significant degradation of Pt/C led to a reduction in the catalyst layer thickness and a decrease in the tortuosity of the ionomer, shortening the ion transport path. Consequently, the ion transport resistance decreased, which is reflected in the reduced proton ion transport resistance observed in the DRT and WLR after degradation.

## 4. Conclusions

The degradation of the binder ionomer was analyzed during the accelerated degradation process of the electrode catalyst. The degradation of the catalyst layer was confirmed through CV, which showed that the ECSA decreased by more than 50% after 20,000 cycles of voltage cycling (0.6–0.95 V). To analyze binder degradation, the DRT method, which can differentiate and measure ORR resistance and proton transport resistance in situ within the catalyst layer, was applied. However, the DRT analysis showed that the ion transport resistance of the binder decreased during the AST process. This result was consistent with the trend observed in the Warburg-like response (WLR), another EIS method. The SEM analysis of the catalyst layer cross-section before and after the catalyst-AST revealed that while Pt and carbon supports degraded, the binder experienced relatively less degradation, leading to improved network formation between binders and better ion transport path formation up to the polymer membrane. During the Pt catalyst-AST process, the DRT and WLR in situ analyses allowed for the measurement of the reduction in catalyst layer thickness and improvement in the binder network, rather than simply focusing on the chemical degradation of the binder.

## Data Availability

The data presented in this study are available on request from the corresponding author.

## Supplementary Materials

The following supporting information can be downloaded at https://www.mdpi.com/article/10.3390/ma17174425/s1, Figure S1. Kramer–Kronigs validation of EIS.
