# Supplementary material for: In Situ Analysis of Binder Degradation during Catalyst-Accelerated Stress Test of Polymer Electrolyte Membrane Fuel Cells
Source: Materials (Basel). 2024 Sep 9;17(17):4425. doi: 10.3390/ma17174425 (PMC11395920; doi:10.3390/ma17174425)
Supplement: Supplementary file 1 [file materials-17-04425-s001.zip › materials-3192834-supplementary.pdf]

Supplementary Material

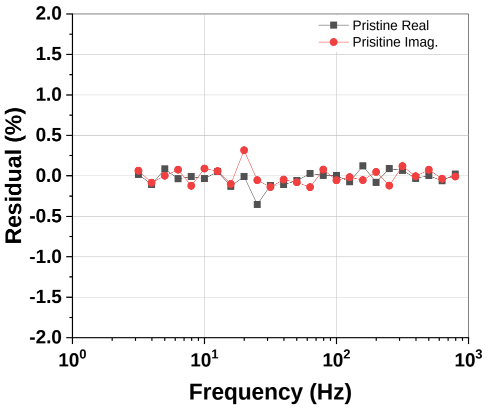

(a)

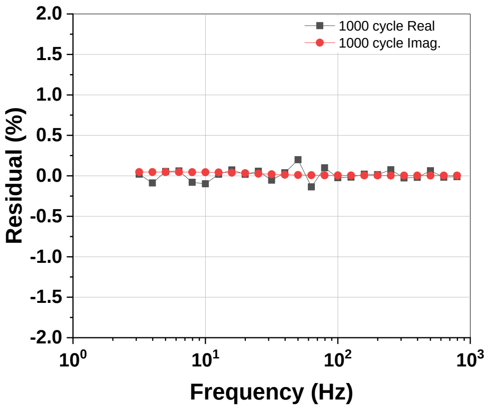

(b)

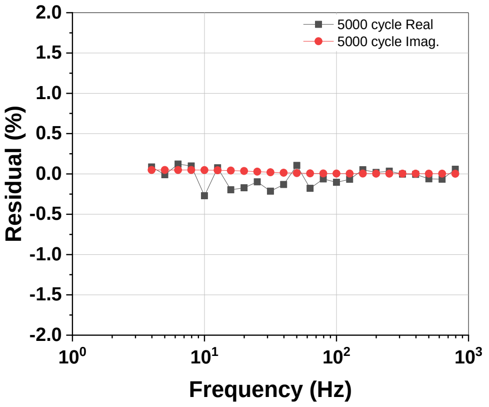

(c)

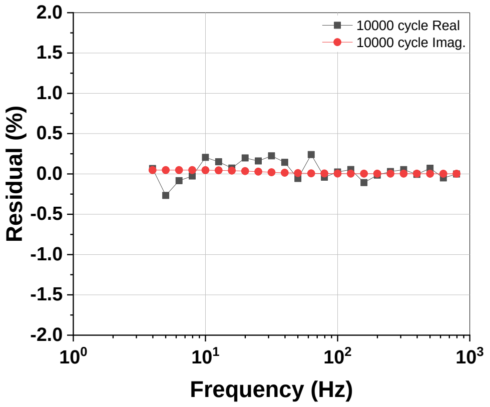

(d)

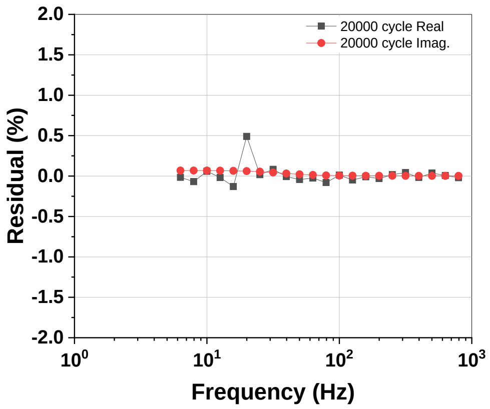

(e)

Figure S1. Kramer-Kronigs validation of EIS after (a) pristine, (b) AST 1000 cycles, (c) AST 5000 cycles, (d) AST 10000 cycles, and (e) AST 20000 cycles, respectively. All plots show much lower residuals than 1 %.
